# In Situ Monitoring and Quantitative Determination of R27 Plasmid Conjugation

**DOI:** 10.3390/life12081212

**Published:** 2022-08-10

**Authors:** Marta Gibert, Carlos J. Jiménez, Jaume Comas, Ellen L. Zechner, Cristina Madrid, Carlos Balsalobre

**Affiliations:** 1 Departament de Genètica, Microbiologia i Estadística, Universitat de Barcelona, Avda, Diagonal 643, 08028 Barcelona, Spain; 2 Laboratori de Citometria/Genòmica CCiT, Parc Científic de Barcelona, Baldiri Reixac 10, 08024 Barcelona, Spain; 3 Institute of Molecular Biosciences, University of Graz, Humboldtstrasse 50, A-8010 Graz, Austria

**Keywords:** plasmid conjugation, IncHI, R27, fluorophores, in situ monitoring, flow cytometry

## Abstract

Horizontal gene transfer (HGT) by plasmid conjugation is a major driving force in the spread of antibiotic resistance among *Enterobacteriaceae*. Most of the conjugation studies are based on calculation of conjugation ratios (number of transconjugants/number of donors) after viable counting of transconjugant and donor cells. The development of robust, fast and reliable techniques for in situ monitoring and quantification of conjugation ratios might accelerate progress in understanding the impact of this cellular process in the HGT. The IncHI1 plasmids, involved in multiresistance phenotypes of relevant pathogens such as *Salmonella* and *E. coli*, are distinguished by the thermosensitivity of their conjugative transfer. Conjugation mediated by IncHI1 plasmids is more efficient at temperatures lower than 30 °C, suggesting that the transfer process takes place during the environmental transit of the bacteria. In this report, we described a methodology to monitor in situ the conjugation process during agar surface matings of the IncHI1 plasmid R27 and its derepressed derivative drR27 at different temperatures. A three-color-labeling strategy was used to visualize the spatial distribution of transconjugants within the heterogeneous environment by epifluorescence and confocal microscopy. Moreover, the fluorescent labelling was also used to quantify conjugation frequencies in liquid media by flow cytometry.

## 1. Introduction

Plasmid conjugation is one of the main mechanisms responsible for horizontal gene transfer (HGT), a driving force for the evolution of bacterial species and promoting adaptation to new environmental niches [1]. From a clinical point of view, the acquisition of a plasmid carrying virulence factors and/or antimicrobial resistance genes might provide competitive advantages for the pathogenic bacteria to successfully cause infection. Incompatibility group HI1 (IncHI1) plasmids contribute to the multiresistance phenotypes of relevant human pathogens [2,3]. These plasmids show a thermosensitive mode of transfer, with an optimal conjugation rate between 22 and 28 °C, and nearly no measurable conjugation at 37 °C [4,5]. This unusual behavior suggests that IncHI1 plasmids might be involved in the dissemination of antibiotic resistances in water or soil environments [6]. In addition to temperature, the physiological state of donor cells also affects conjugation rates; for example, exponential phase of growth stimulates transfer efficiency [7]. In the R27 plasmid—the prototype of the IncHI plasmids—a regulatory circuit, comprising two activators (TrhR and TrhY) and one anti-activator protein (HtdA) is involved in the thermoregulation and physiological control of R27 conjugation [5,8,9,10].

Most studies on plasmid conjugation are based on the calculation of conjugation frequencies after viable counting of transconjugant and donor cells, obtained by antibiotic selection on agar plates. Advances in reporter gene technology provided new insights into the dynamics and extent of spatial dissemination of conjugative plasmids in vitro and in natural environments [10]. The integration of genes encoding reporter proteins such as green fluorescent protein in a conjugative plasmid permits in situ monitoring of the plasmid spread in bacterial communities as monitored in agar-surface grown colonies [11,12,13] or biofilms [14,15]. To monitor in situ conjugation of R1 plasmid and its derepressed derivative R1drd19, a triple-labelling strategy was used [16]. In this work, this triple-labeling strategy has been applied to monitor the conjugation of the R27 plasmid in situ by epifluorescence and/or confocal microscopy. Donor cells carry a conjugative plasmid in which the expression of a cyan fluorescent protein (CFP*) is under the control of the *lac* promoter. Donor cells express a chromosomally encoded LacI repressor while recipient cells lack a functional LacI protein. Therefore, CFP* is only expressed when the conjugative plasmid is transferred into a recipient cell (blue fluorescence). Differentiation between donor and recipient cells is possible due to differential color-labeling. Donor cells are labeled with a plasmid-encoded red fluorescent marker (DsRed2.T3) and recipient cells express a chromosomal gene coding for the yellow fluorescent protein (YFP*). In situ monitoring of R27 conjugation showed a discrete invasion of the plasmid into recipient colonies and also enabled the thermosensitivity of the R27 conjugation to be visualized. Moreover, we demonstrated that this labelling strategy could be easily used as an easy, quick and robust methodology to quantify conjugation ratios using flow cytometry analysis.

## 2. Materials and Methods

### 2.1. Bacterial Strains, Plasmids and Growth Conditions

*Escherichia coli* strains and plasmids used in this study are listed in Table 1. Bacteria were routinely grown in LB (10 g/L NaCl, 10 g/L tryptone, 5 g/L yeast extract). For conjugation experiments, strains were grown in PB medium (1.5 g/L meat extract, 1.5 g/L yeast extract, 5 g/L peptone, 1 g/L glucose, 3.5 g/L NaCl, 1.32 g/L KH2PO4, 4.82 g/L K2HP4·3H2O). For genetic techniques involving λ red recombination strains were grown in 2xTY (2× tryptone-yeast extract: 10 g/L NaCl, 20 g/L tryptone, 10 g/L yeast extract). When needed, selective media contained antibiotics in the following concentrations: kanamycin (Km) 50 µg/mL, chloramphenicol (Cm) 20 µg/mL, ampicillin (Amp) 100 µg/mL, tetracycline (Tc) 15 µg/mL, nalidixic acid (Nal) 30 µg/mL.

### 2.2. Genetic Techniques

Two R27 derivative plasmids, R27-cfp* and drR27-cfp* were generated as previously described [20]. These plasmids encode a Cyan Fluorescent Protein (CFP*) with a point mutation that changes its second amino acid to improve its fluorescence. The cfp* gene fused to a Cm resistance cassette from plasmid pAR92 was inserted into the Tc resistance cassette of R27 and drR27 plasmids. The cfp* gene, under the control of the *lac* promoter, was attached to a Cm resistance cassette from plasmid pAR92 and this construct inserted into the Tc resistance cassette of R27 and drR27 plasmids. The genetic construct cfp*—Cm resistance cassette was amplified from plasmid pAR92 using primers MG01Dca (5′AGCTTTCCCCTTCTAAAGGGCAAAAGTGAGTATGGTGCCTAGGTATTTCACACCGCATAGC3′) and MG02Uca (5′AACCGAACCACTTCACGCGTTGAGAAGCTGAGGTGGTATCGCAAGAATTGCCGGCGGAT3′), that introduce at each end 41 bp homologous to the *tetA* gene of R27. The resulting PCR fragment was electroporated into DY330Nal cells (harboring either R27 or drR27 plasmids) in which the expression of λ red recombinase had been temperature induced. The cells were incubated at 30 °C for 60–90 min prior plating on 2xTY plates supplemented with Cm for selection of recombinants. The obtained clones were tested by PCR using the primers DcaUp (5′CATCGCGATGACTTAGTAAAG3′) and UcaDown (5′CTATTAAACCAAGCCCAAAAC3′). The plasmids R27-cfp* and drR27-cfp* were transferred by conjugation from DY330Nal strain to the final donor strain, SAR08. No cfp* expression was detected since SAR08 cells constitutively express the LacI repressor.

For labeling the SAR08 strain, plasmid pAR179Km, expressing red-fluorescent DrRed2.3 under the control of a ribosomal promoter was constructed. To achieve this, plasmids pAR179 and pAR65 were digested using *Bam*HI restriction enzyme and the generated fragments containing the DsRed2.T3 and Km resistance sequences were purified and ligated. To enable the three-color labeling, the non-transferable low copy plasmid pAR179Km was electroporated into the SAR08 donor cells.

### 2.3. Conjugation Assays

Agar surface matings for in situ monitoring were prepared as described previously [16]. Strain SAR08 (pAR179Km), expressing red fluorescence, and strain SAR20, labeled by a chromosomal insertion of the gene coding for the YFP* fluorescent protein (yellow), were used as donor and recipient cells, respectively. Sixteen-hour (O/N) cultures of the donor and recipient strains in PB media, supplemented with the required antibiotics, were washed and diluted in NaCl 0.9% to an OD_600nm_ of 0.2. Dilutions 10^–3^ and 10^–4^ of donor and recipient cells, respectively, were spread on LB agar plates and incubated at the indicated temperature. Fluorescence microscopy and conjugation frequency determination by viable count were performed after 18 h of incubation at 37 °C or after 36 h when incubated at 25 °C or 30 °C. Mating mixtures on plates were incubated for longer time that standard protocols for mating in liquid media (see below) to allow colony formation and contact between donor and recipient colonies. When incubated at 37 °C, the incubation was lower than at 25 °C/30 °C, by differences in growth kinetics. For samples analyzed by confocal laser microscopy, the same dilutions of the donor and recipient suspensions were spread on a microscope glass slide covered with 1 mL of LB agar.

For flow cytometry quantification and conjugation frequency determination by viable count, mating assays in liquid media were performed as previously described [9]. Cultures of donor and recipient strains were grown in PB supplemented with the required antibiotics at 25 °C under static conditions for 16 h. Then, cells were washed with PB to eliminate the antibiotics and 0.4 mL of the recipient strain suspension and 0.1 mL of the donor strain suspension were added to 1 mL of PB and incubated at 25 °C for 2 h. To analyze drR27 conjugation by flow cytometry throughout the growth curve, the same protocol was used with PB cultures of donor and recipient strains at 25 °C under shaking, as previously described [8].

### 2.4. Microscopy

Epifluorescence microscopy was performed using a Zeiss Axioskop epifluorescence microscope equipped with a mercury vapor lamp (HBO50, OSRAM) and Chroma filter sets DsRed, EGFP, ECFP, and EYFP (Chroma Techn. Corp., Bellows Falls, VT, USA). Pictures were acquired using a VISICAM camera (Visitron Systems, GmbH., Germany) and the MetaMorph^®^ Imaging Package 4.0 (Molecular Devices Corporation, San José, CA, USA). Confocal laser scanning microscopy (CLSM) was performed with a LEICA AOBS SP2 MP microscope using appropriate laser settings for simultaneous monitoring of CFP*, YFP* and Dsred2.T3 fluorophores. Z-stacks of overlay images representing all three detection channels were processed to video sequences. Obtained images were processed using Irfanview and LAS-AF-Lite 2.6.0 software (Leica Microsystems, Wetzlar, Germany). In all samples, a minimum of six different fields were observed.

### 2.5. Flow Cytometry Assays

To determine conjugation ratios by flow cytometry, the mating mixture was diluted 1/100 in filtered Ringer ¼ solution and kept on ice. Samples were analyzed in the CCiT (Parc Científic de Barcelona) using a Gallios multi-color flow cytometrer instrument (Beckman Coulter, Inc., Fullerton, CA, USA) set up with the 3-lasers 10 colors standard configuration. Excitation of the sample was performed using the blue (488 nm) and violet (405 nm) laser. Forward scatter (FS), side scatter (SS) and fluorescence values were collected using logarithmic scales. FS was used as discriminating parameter. The single-cell bacterial population was selected on a forward-side scatter scattergram. Debris (from culture media and dead cells) and aggregates were excluded in this graph. Fluorescence emitted by YFP* (excitation 488 nm) was collected using a 525/40 band-pass filter. Emission from DsRed2.T3 was collected through a 620/30 nm filter. A 450/40 nm filter was used to collect fluorescence from CFP* (excitation 405 nm).

### 2.6. Statistical Analysis

Differences between average values were tested for significance by performing an unpaired two-sided Student’s test. The levels of significance of the resulting *p* values are indicated in the figure legends.

## 3. Results and Discussion

### 3.1. In Situ Monitoring of the R27 and drR27 Conjugation

**Figure 1:**
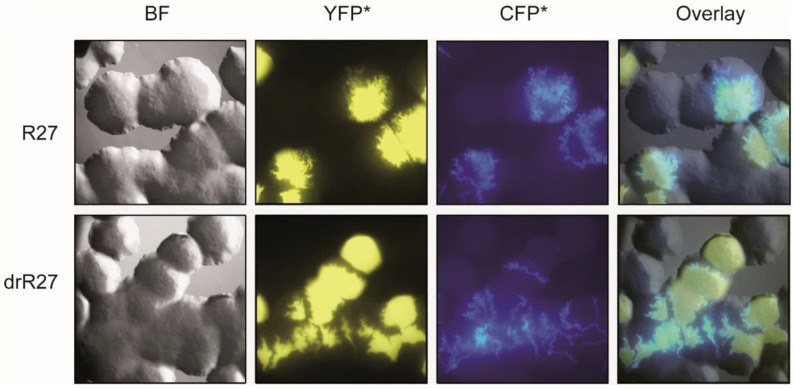
In situ monitoring of the R27 and drR27 transfer on solid surfaces using epifluorescence microscopy. Columns represent bright field (BF), epifluorescence (YFP* for recipient cells and CFP* for transconjugants), and overlay images of representative areas of agar surfaces inoculated with strain SAR08/pAR179Km carrying the R27 plasmid (upper row) or the drR27 plasmid (lower row) as a donor cells, and SAR20 as a recipient, after 36 h of incubation at 25 °C.

To monitor the in situ transfer of IncHI1 plasmids between *E. coli* cells, derivatives of R27 and drR27 plasmids carrying a cyan fluorescence reporter gene (cfp*) were generated. drR27 is a derepressed R27 variant due to a mutation in the plasmid encoded *htdA* gene that causes an increase on the transcription of *tra* genes and, consequently, a drastic increase in the conjugation frequency. In order to monitor the conjugation, the strain SAR08 carrying the pAR179Km plasmid, that express constitutively a red fluorescent protein, DsRed2.T3, was used as donor and the strain SAR20, labeled with yellow fluorescence protein (YFP*) as recipient. To efficiently discriminate between donors and transconjugants, the cfp* gene is only expressed in transconjugant cells since the donor cells carry a LacI repressor that maintain the cfp* gene in a silenced state. Conjugation on solid surfaces was tested by inoculating both donor and recipient cells on LB agar plates and incubation for 36 h at the permissive temperature for conjugation (25 °C). The bacterial populations were observed under epifluorescence microscopy (Figure 1). The epifluorescence microscope allows for the detection of the YFP* (yellow labelled, indicating recipient cells) and CFP* (blue labelled, indicating transconjugant cells) fluorophores but not the DsRed2.T3 fluorophore. However, donor colonies can be identified with the bright field image. Transconjugant cells were located throughout the contact surface between a donor and a recipient colony for both wt and derepressed plasmids (Figure 1). The drR27 is transferred at 25 °C with a frequency higher than four log-fold as compared to R27 during mating assays in liquid media [8,23]. However, apparently identical images were detected when the donor cells carried either R27 or drR27. No important differences in the thickness of the layer corresponding to transconjugant cells were detected by visual observation (Figure 1).

**Figure 2:**
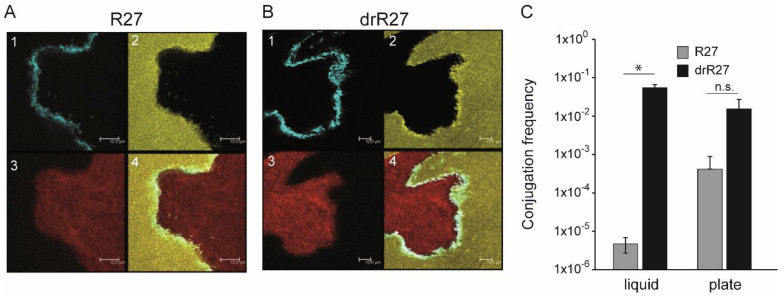
Confocal microscopy reveals the single cell level spatial organization of R27 (**A**) and drR27 transconjugants (**B**) emerging during mating on agar surfaces. The epifluorescence channels: CFP*, corresponding to the transconjugants (**A1**,**B1**), YFP*, corresponding to the recipient cells (**A2**,**B2**) and Dsred2.T3, corresponding to the donor cells (**A3**,**B3**), are shown. The representative mating areas are also displayed as overlay images (**A4**,**B4**). Images were taken on representative areas of agar surfaces inoculated with *E. coli* SAR20 as a recipient and SAR08/pAR179Km donor cells carrying plasmids R27 or the drR27, after 36 h of incubation at 25 °C. The scale bars represent 100 µm. (**C**) Frequency of conjugation of R27 and drR27 plasmids determined in liquid conjugation assays or on agar surface (plate) mating assays at 25 °C. Mean values of three independent experiments with standard deviations are plotted. Statistical differences between average values were tested by performing an unpaired, two-tailed Student’s *t* test. The resulting *p*-values are presented by the following symbols: n.s. = non-significant (*p* > 0.05); * *p* < 0.05.

To reveal the extent of plasmid invasion into recipient colonies with greater precision, the depth of transconjugant cell layers in the interphase between donor and recipient colonies was visualized with agar surface matings by confocal laser scanning microscopy (CLSM) (Figure 2). CLSM resolution supports visualization of single cells: recipient (yellow), transconjugant (cyan), and donor cells (red) (Figure 2). In the confluence between donor and recipient colonies, a discrete layer of transconjugants cells was observed, with a width limited to a few cells. These data are consistent with a limited ability of the conjugative plasmids to penetrate into the recipient population. One might expect that once a recipient cell receives a conjugative plasmid, becoming a transconjugant, it will be able to quickly act as a donor and promote transfer of the plasmid received to neighboring recipient cells. However, that is not what we detect with R27 and drR27 plasmids under the mating conditions used. The limited invasion observed for the IncHI1 plasmid into recipient colonies has also been described for conjugative plasmids from very diverse compatibility groups, such as IncP, IncF, IncI and IncW [11,16]. The specific reason for the reported low penetrability of conjugative plasmids on solid media mating assays is uncertain. Nutrient limitation in densely packed colonies might affect conjugation. Hence, it has been described with IncP plasmids that replenishing nutrients increased the extent of plasmid invasion on surfaces [13]. Spatial constraints and motility might also be important factors to consider. Potential pairs need some degree of movement for plasmid transfer even on surfaces and in biofilms. Motility downregulation by carrying IncF plasmid was proposed to be related with the low spread of plasmid s into the recipient colony [16].

As depicted by CLSM, the amount of transconjugant cells generated during mating assays with drR27 seems to be slightly higher than for R27 plasmid. The observed difference is negligible, however, when compared with the conjugation frequencies for both plasmids in liquid culture, i.e., more than four log-fold higher for drR27 than for the R27 plasmid (Figure 2C). Conjugation frequencies for R27 and drR27 plasmids on solid media (agar plates) were also determined using the classical methodology based on plating and viable cell count after mating incubation. Consistent with the in situ monitoring experiment, the great difference in the conjugation frequencies detected in liquid drops when mating assays were performed on solid media (Figure 2C).

**Figure 3:**
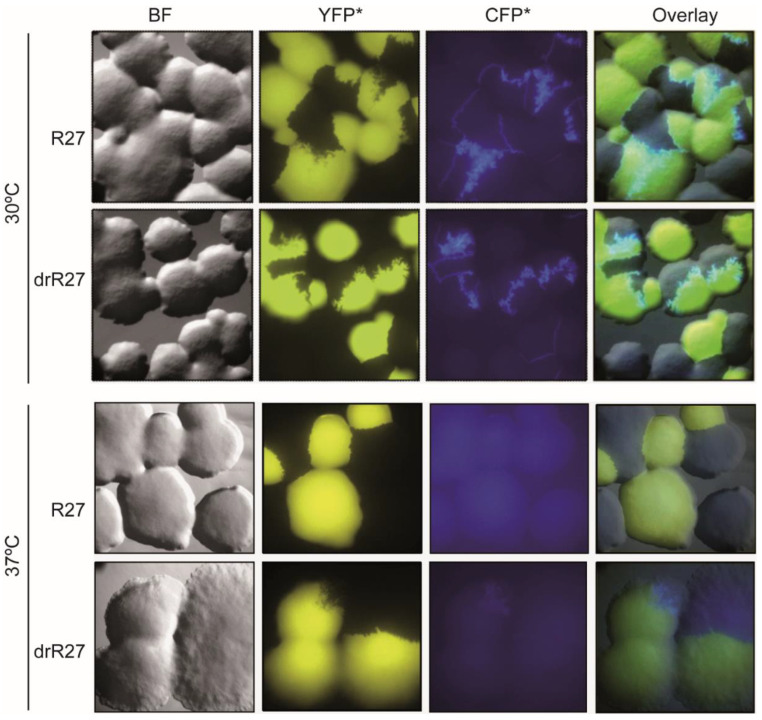
In situ monitoring of the R27 and drR27 transfer on solid surfaces at 30 °C and 37 °C using epifluorescence microscopy. Columns represent bright field (BF), epifluorescence (YFP* for recipient cells and CFP* for transconjugants), and overlay images of representative areas of agar surfaces inoculated with *E. coli* SAR20 recipient and SAR08/pAR179Km donor cells carrying the R27 plasmid (upper row) or the drR27 plasmid (lower row) after 36 h of incubation at 30 °C or 18 h of incubation at 37 °C.

**Figure 4:**
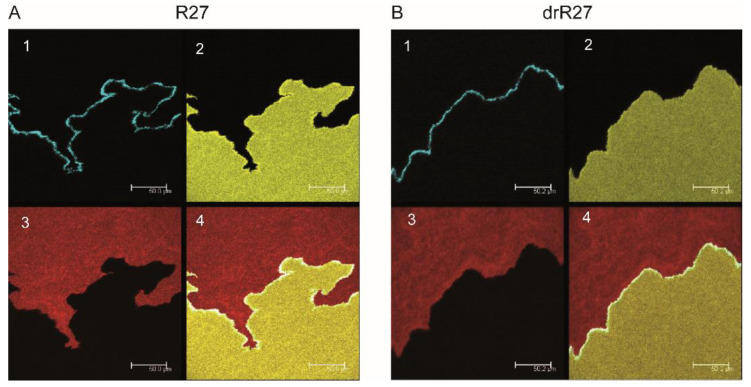
Confocal laser scanning microscopy (CLSM) of agar surface matings performed at 30 °C of R27 (**A**) and drR27 transconjugants (**B**). The epifluorescence channels: CFP*, corresponding to the transconjugants (**A1**,**B1**); YFP*, corresponding to the recipient cells (**A2**,**B2**) and Dsred2.T3, corresponding to the donor cells (**A3**,**B3**), are shown. The representative mating areas are also displayed as overlay images (**A4**,**B4**). Images were taken on representative areas of agar surfaces inoculated with *E. coli* SAR20 recipient and SAR08/pAR179Km donor cells carrying the R27 or the drR27 plasmids after 36 h of incubation at 30 °C. The scale bars represent 50.0 µm (**A**) and 50.2 µm (**B**), respectively.

A hallmark of IncHI1 plasmids is thermosensitive conjugation [4]. In situ monitoring was next used to observe mating mixtures on solid surfaces at 30 °C and 37 °C. At 30 °C (Figure 3), a continuous line of transconjugants between donor and recipient colonies was observed, similar to the results at 25 °C (Figure 1). Again, no clear differences were detected between mating mixtures of R27 and its derepressed derivative drR27 on solid media using epifluorescence (Figure 3). These results were confirmed using CLSM (Figure 4).

**Figure 5:**
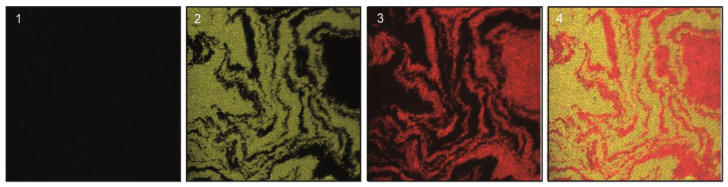
CLSM of agar surface mating performed at 37 °C. The epifluorescence channels CFP*, corresponding to the transconjugants (**1**); YFP*, corresponding to the recipient cells (**2**) and Dsred2.T3, corresponding to the donor cells (**3**), are shown. The representative mating areas are also displayed as overlay images (**4**). Images were taken of a representative area of an agar surface inoculated with *E. coli* SAR20 recipient and SAR08/pAR179Km donor cells carrying the drR27 plasmids, after 18 h of incubation at 37 °C.

At 37 °C, the amount of transconjugants detected by epifluorescence microscopy dropped drastically (Figure 3), consistent with previous data on the thermosensitivity of R27 conjugation in liquid cultures [4,5]. In the case of the R27 plasmid conjugation, no transconjugant cells were observed at 37 °C in any assay replica. With drR27, transconjugants could be visualized, although very rarely, suggesting that the transfer of drR27 plasmid occurs at a very low frequency. The conjugation ratio and the amount of transconjugants at 37 °C were so low that CLSM did not allow for the detection of transconjugant cells with either R27 or drR27 (Figure 5).

### 3.2. Use of Flow Cytometry to Determine Conjugation Frequencies

**Figure 6:**
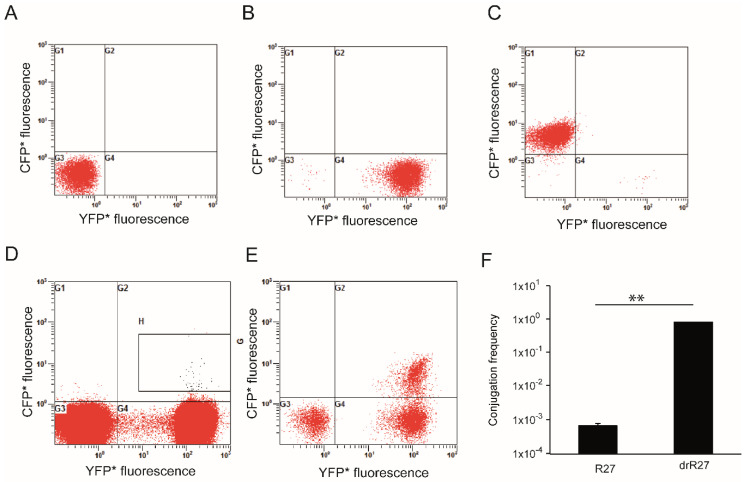
Using flow cytometer to quantify R27 transfer. Detected fluorescence by the cytometer using pure cultures of the donor strain (SAR08/pAR179Km), where no fluorescence is detected (**A**); the recipient strain (SAR20), emitting yellow fluorescence (**B**) and the strain DY330Nal carrying the plasmid drR27-cfp* (a Δ*lacI* strain which therefore allows the expression of cfp* present in the conjugative plasmid and that, dissimilar from the recipient strain, is not marked with YFP*) (**C**). Analysis of the conjugation frequency at 25 °C obtained using stationary phase donor cultures of SAR08/pAR179Km carrying R27 (**D**) or drR27 (**E**) plasmids, respectively. G3 quadrant shows the quantified donor cells, G4 quadrant shows the recipient cells and G2 quadrant shows the transconjugants. Selection within the H region was performed due to the low frequency of conjugation or the R27 plasmid, in order to ensure recipient cells are not been wrongly selected as transconjugants in that case. (**F**). Frequency of conjugation of R27 and drR27 plasmids in liquid medium at 25 °C, calculated by flow cytometry. Mean values of three independent experiments with standard deviations are plotted. Statistical differences between average values were tested by performing an unpaired, two-tailed Student’s *t* test. The resulting *p*-value is presented by the following symbol: ** *p* < 0.005. All bacterial cultures were grown in PB at 25 °C under static conditions.

Based on the differences in the fluorescence depicted by donor, recipient and transconjugant cells, flow cytometry was applied in order to directly quantify the amount of transconjugant cells in a population. To set up the flow cytometry methodology non-stained cells were used to establish the gates corresponding to bacterial populations. Then, cultures of cells expressing a single fluorescent protein were used to determine compensation values in multiple-fluorescence detection (Figure 6A–C). The excitation of DsRed2.T3 at 448 nm was suboptimal, causing a poor resolution of cells emitting red fluorescence. However, the donor cell population was characterized by being non-YFP/non-CFP (G3 quadrant) (Figure 6A). CFP* and YFP* expressing cells were clearly distinguished, by localizing in G1 and G4 quadrants, respectively (Figure 6B,C). Transconjugants, which are characterized by expressing CFP* and YFP*, localize in the G2 quadrant of the graph (Figure 6E).

Liquid cultures of the donor strain with either R27 or drR27 plasmid grown at 25 °C for 16 h were used for mating experiments. Conjugation mixtures were analyzed after 2 h mating incubation. The cell distribution for conjugation of R27-cfp* and drR27-cfp* is shown in Figure 6D,E, respectively, and the conjugation frequency was calculated as the ratio between donor and transconjugant cells (Figure 6F). The conjugation frequency of drR27 plasmid at 25 °C reaches levels of about 1, whereas when using R27 plasmid, conjugation frequency decreases more than three log-fold. These frequencies are in good agreement with those calculated by traditional antibiotic plating assays ([8] and Figure 2C). Flow cytometry is a faster method to determine frequency of plasmid conjugation. However, a weak point of this methodology is that its reliability is compromised when plasmids with very low frequency of conjugation are tested. For instance, when using the R27 plasmid, the determination of conjugation frequencies is compromised due to the very low relative amount of transconjugant cells. In the samples from R27 mating assays, the percentage of transconjugants is so low that technical background noise can importantly affect the output of transconjugant quantification.

It should be noted that in studies of plasmid conjugation, derepressed derivatives of conjugative plasmids are often used to facilitate transconjugant detection independently of the methodology used for quantification. For instance, most studies with the F plasmid of *E.coli* or the pSLT plasmid of *Salmonella* have been performed using derepressed variants [24,25,26].

### 3.3. Use of Flow Cytometry to Monitor the Frequency of drR27 Conjugation through the Growth Curve

**Figure 7:**
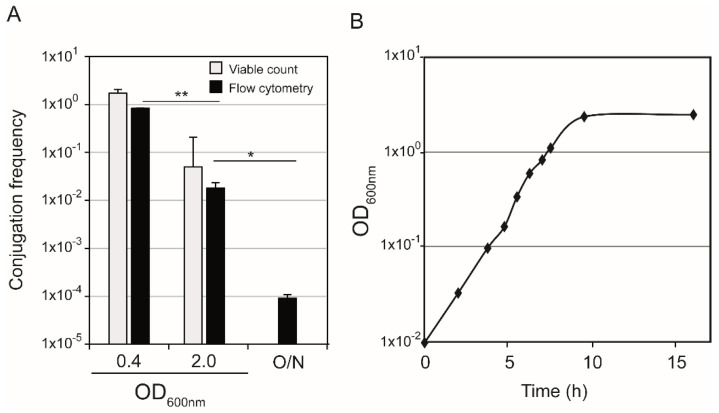
Conjugation frequencies of the plasmid drR27 calculated by flow cytometry assay and traditional plate-based conjugation experiments, using donor cells at different time points during the growth curve (**A**). The OD_600nm_ of the cultures at 25 °C of the donor cells are indicated. O/N indicate cultures in late stationary phase (16 h incubation). Statistical differences between average values calculated by flow cytometry were tested by performing an unpaired, two-tailed Student’s *t* test. The resulting *p*-values are presented by the following symbols: * *p* < 0.05; ** *p* < 0.005. Growth curve of donor strain (**B**), a representative experiment is shown. All bacterial cultures were grown in PB at 25 °C under shaking conditions.

R27 conjugation is promoted when donor cells are in logarithmic phase of growth but it severely drops when they enter stationary phase [7]. To further test the suitability of flow cytometry-based quantification of conjugation rates, this methodology was applied to study the effect of the growth phase on conjugation, using the derepressed drR27 plasmid. The conjugation frequency was determined with donor cells in different phases of growth (Figure 7).

Highest frequency of conjugation was observed when donor cells were in mid-exponential phase of growth (OD_600nm_ of 0.4). Conjugation frequencies dropped when donor cells from cultures with higher OD_600nm_ were used. Hence, a more than 1 log-fold decrease is observed when using donor cultures entering stationary phase (OD_600nm_ of 2.0) and at late stationary phase (16 h incubation, O/N), the conjugation frequency drops almost four log-fold log, as compared to exponentially growing donor cells. The observed increase in the conjugation frequency in logarithmic phase is in agreement with the results obtained by plating based quantification of transconjugant and donor cells at mid-exponential phase and in the entry to stationary phase (light grey bars in Figure 7, [7]).

## 4. Conclusions

Plasmid conjugation plays a relevant role in the spread of antibiotic resistance and bacterial evolution. Considering the serious increase in antibiotic resistance and the appearance of new pathogenic variants, it is crucial to fully understand the process of plasmid conjugation. Therefore, efforts to describe environmental factors that promote or repress plasmid conjugation, or to test the efficiency of certain molecules to inhibit plasmid transfer can be of critical relevance towards development of containment measures for transfer of plasmids between bacteria. Robust and efficient methodologies to quantify conjugation frequencies and to monitor in situ plasmid conjugation are necessary. Use of fluorescent reporter genes is an efficient strategy to detect and quantify separately donor, recipient and transconjugant cells during mating assays and, therefore, may be applied to study plasmid conjugation. Such an experimental strategy has been previously applied with plasmids from the incompatibility groups IncP, IncF, IncI and IncW. In this report, fluorescent reporter genes have been used for the first time to monitor conjugation of plasmids from the IncH incompatibility group, a low copy number and wide host range plasmids that are considered the main carrier of multiple resistance in *Salmonella* Typhi [27]. The three-color-labelling strategy used was previously applied to monitor F plasmid conjugation [16]. In this report, in addition to monitoring plasmid conjugation in situ, using epifluorescence microscopy and CLSM, we also quantify the three types of cells (recipient, donor and transconjugant) involved in mating assays by using flow cytometry. The determination of conjugation ratios by flow cytometry is an advantage of the three-color- based strategy as compared to previous approaches, combining fluorescence and luminescence, used to efficiently detect conjugation in situ [11]. Bacterial cell counting by flow cytometry provides a significant advantage in both speed and accuracy over the traditional plate-based viable counting method to determine conjugation frequencies with plasmids showing high frequencies of transfer. Flow cytometry is thus a robust approach to screen different parameters that might affect frequency of conjugation, as shown in this report by determination of drR27 conjugation through the growth curve.

## Figures and Tables

**Table 1:** Strains and plasmids used in this work.

Strain/Plasmid	Description	Reference
MG1655	F-, *ilvG*, *rphI*	[17]
AAG1	MG1655 Δ*lacZYA*	[18]
SAR20	CSH26 *attBλ::bla*-P_A1/04/03_-yfp*-To, Amp^R^	[19]
SAR08	CSH26 *attBλ::bla*-*lacI*^Q1^, Amp^R^	[16]
DY330Nal	W3110 Δ*lacU169* λcI857 Δ(*cro-bioA*), Nal^R^	[20]
R27	IncHI1 Tc^R^	[21]
drR27	R27 *htdA*::IS10	[8]
R27-cfp*	R27 *tet::cat* PA1/04/03-cfp*-To	This work
drR27-cfp*	R27 *htdA*::IS10 *tet::cat* PA1/04/03-cfp*-To	This work
pAR92	*cat*-P_A1/04/03_-cfp*-T0 cassette, Amp^R^, Cm^R^	[20]
pAR179	P*_rrnB_*_-P1_-*dsred2.T3*–T0 cassette, Cm^R^	[22]
pAR65	Km^R^ vector	Reisner et al. unpublished
pAR179Km	pAR179 Cm^S^ Km^R^	This work

## Data Availability

Not applicable.
